# Development and psychometric evaluation of an assessment tool assessing Pharmacists’ knowledge and practice on antimicrobial prophylaxis among cancer patients

**DOI:** 10.1371/journal.pone.0356508

**Published:** 2026-08-24

**Authors:** Lee Jia Hui, Nicole Lam Koh Yie, Justine Yap, Muhamad Nasrul, Ali Haider Mohammed, Bassam Abdul Rasool Hassan, Lee Jia Jia, Dinesh Sangarran Ramachandram, Ruth Sim, Angelina Lim, Juman Dujaili, Ali Blebil

**Affiliations:** 1 School of Pharmacy, Monash University Malaysia, Jalan Lagoon Selatan, Subang Jaya, Selangor, Malaysia; 2 Faculty of Pharmacy, Al Rafidain University, Baghdad, Iraq; 3 Faculty of Pharmacy and Pharmaceutical Sciences, Monash University, Parkville, Victoria, Australia; 4 Murdoch Children’s Research Institute, Royal Children’s Hospital, Parkville, Victoria, Australia; 5 Pharmacy Department, Medical School, Swansea University, Swansea, United Kingdom; Curtin University Bentley Campus: Curtin University, AUSTRALIA

## Abstract

**Background:**

Despite advancements in cancer therapy, opportunistic infections remain a significant obstacle to successful patient outcomes. This highlights the necessity for research into the role of antimicrobial prophylaxis in preventing opportunistic infections within oncology care. Therefore, this study aims to develop and validate a questionnaire that assesses pharmacists’ knowledge and practices regarding antimicrobial prophylaxis in cancer patients.

**Method:**

The research was conducted in 2 phases. Phase I focused on questionnaire development, which included assessing content validity using face validity, Delphi technique, and pilot testing. An initial questionnaire comprising 53 items was generated. The questionnaire was then distributed, and the data were collected from Survey Monkey. Phase II involved the psychometric evaluation of the questionnaire’s validity and reliability. Data analysis was performed using Item Response Theory (IRT), Exploratory Factor Analysis (EFA), and Cronbach’s alpha to establish construct validity and reliability.

**Result:**

The finalized questionnaire consists of 43 items, comprising 29 knowledge and 14 practice items. IRT analysis was applied to knowledge items, with acceptable discrimination (≥0.25), difficulty (−3–3), and guessing (≤0.4) parameters. Eight items were removed from the knowledge domain based on IRT analysis. Furthermore, EFA has shown high correlation between items and their respective factor, with each receiving an acceptable factor loading of >0.4. However, one domain has a reliability value of <0.6, hence, the domain was removed from the practice domain. Overall, Cronbach’s alpha of 0.83 indicates strong internal consistency among the questionnaire items.

**Conclusion:**

The results of this study have validated that the developed questionnaire possesses exceptional psychometric qualities, confirming its accuracy and reliability to evaluate pharmacists’ knowledge and practice towards antimicrobial prophylaxis in oncology care. The validated tool enables future research and targeted educational interventions to improve pharmacists’ skills in managing infection risks for cancer patients.

## 1. Introduction

Cancer remains a significant global health concern, with an estimated 20 million new cases and almost 10 million cancer-related deaths worldwide in 2020 [1]. Despite advanced cancer treatment, infection continues to be the major barrier to effective cancer treatment. Aggressive cancer therapies compromise the immune system by suppressing white blood cell production. Recent evidence indicates that neutropenia is a significant adverse event associated with both conventional and novel anticancer agents [2], and is frequently observed following chemotherapy in real-world cohorts, where it is linked to serious infections and adverse clinical outcomes [3]. This highlights the importance of antimicrobial prophylaxis in preventing opportunistic infections. Recent systematic review shows antibiotic prophylaxis effectively reduces infection rates in oncology settings; However, the increased risk of antimicrobial resistance necessitates a careful balance between prevention benefits and the implementation of antimicrobial stewardship [4].

Pharmacists play a role in tailoring antimicrobial prophylaxis to prevent antimicrobial resistance. Pharmacists are involved in identifying drug-related issues. A study in Iran identified 1281 drug-drug interactions (DDIs) among 200 patients with hematologic malignancies, prompting 1059 interventions by clinical pharmacists [5]. Pharmacists also contribute to the future development of cancer treatment and prophylactic regimens. Nelson et al. emphasized that pharmacists are involved in research to elucidate the pharmacokinetics of renally eliminated drugs in cancer patients with augmented renal clearance [6]. However, inadequate pharmacist knowledge can lead to inappropriate antibiotic selection, delays in administration, and prolonged antimicrobial therapy. Elshenawy et al. conducted a cross-sectional descriptive study among pharmacists to evaluate their knowledge, attitudes, and perceptions regarding antibiotic resistance and antimicrobial stewardship in a UK NHS Foundation Trust [7]. One aspect assessed among pharmacists was their understanding of antimicrobial stewardship principles and appropriate antibiotic use. The findings revealed notable gaps in pharmacists’ knowledge and awareness, which may contribute to suboptimal antimicrobial management and potentially increase the risk of antimicrobial resistance in clinical practice.

There is limited research on pharmacists’ knowledge and practice of antimicrobial prophylaxis in cancer patients. Existing studies have mainly focused on antimicrobial stewardship in oncology care, with little attention to prophylaxis specifically [8]. Consequently, there is a lack of validated tools to evaluate pharmacists’ knowledge and practice in this area. This study addresses this gap by developing and validating a questionnaire to assess pharmacists’ knowledge and practice regarding antimicrobial prophylaxis in oncology. The findings aim to identify deficiencies, support improvements in safe and effective prophylactic practices, and serve as a foundation for future educational programs to strengthen pharmacists’ roles in cancer care.

## 2. Methods

### 2.1. Study design

A cross-sectional survey was selected as the instrument of choice to develop and validate the questionnaire assessing pharmacists’ knowledge and practice towards antimicrobial prophylaxis among cancer patients. It was conducted in two phases, and in the first phase, questionnaire items were developed and refined through two rounds of expert review using the Delphi technique. The second phase involved psychometric testing of the items’ validity and reliability. Questionnaire development was carried out from February to March 2025, followed by data collection from 1^st^ of April to 3oth June 2025. Data analysis was conducted in July 2025, after sufficient responses had been collected.

### 2.2. Study setting

The research was conducted in Malaysia, which has a diverse range of pathogens influenced by its tropical climate and multicultural society, making it valuable to study the antimicrobial management pattern. Malaysia has a notable increase in cancer and antimicrobial resistance cases as well [9].

### 2.3. Study population

Inclusion criteria include Fully Registered Pharmacists (FRP) working in Malaysian hospitals. The exclusion criteria are to exclude Provisionally Registered Pharmacists (PRP) and the FRP working outside of hospital settings to ensure the accuracy of the results.

### 2.4. Endpoints/outcomes

The main outcome is to develop and validate a questionnaire assessing pharmacists’ knowledge and practice on antimicrobial prophylaxis among cancer patients.

### 2.5. Study instrument

The study employed a multi-phase design to assess the psychometric properties of the items: Phase I: Questionnaire development, and Phase II: Psychometric evaluation of the questionnaire.

#### 2.5.1. Phase I: Questionnaire Development.

An extensive literature review was carried out using reputable research databases like OvidMedline, PubMed, and Scopus. The knowledge section of the questionnaire was designed to focus on the domains of infections in cancer patients, antimicrobial stewardship (AMS), and antimicrobial resistance (AMR), pharmacists’ role in cancer care, infection prevention in cancer patients, and lastly, patient safety and error prevention in prophylactic antimicrobial use. The practice section of the questionnaire was developed using domains of common antimicrobial prophylaxis recommendations in cancer patients, management of drug-drug interaction (DDI), and monitoring with follow-up of the antimicrobial prophylaxis in cancer patients [10–16].

To assess content validity, two rounds of the Delphi technique have been run with five experts in the clinical field. The panel consisted of 5 members: 2 pharmacists who were academicians, 1 oncology pharmacist, 1 oncologist who was also a microbiologist, and 1 antimicrobial pharmacist. The questionnaire was formulated in the form of degree of relevance, with a scale of 1 (least relevant) – 4 (most relevant). Only the items with an item-level content validity index (I-CVI) value of at least 0.87 were accepted [17]. The items were amended according to the experts’ responses, and a total of 53 items of the questionnaire were finalised.

Face validity was evaluated by 10 hospital pharmacists, followed by pilot testing with 30 hospital pharmacists, aiming to identify potential issues with the clarity and feasibility of the questionnaire before the data collection. Participants reviewed each item, provided comments, and highlighted any uncertainty. Analysis of the feedback indicated that no revisions were required, and the questionnaire could be completed in approximately 15 minutes. The questionnaire was finalised and prepared for the data collection.

**Fig 1:**
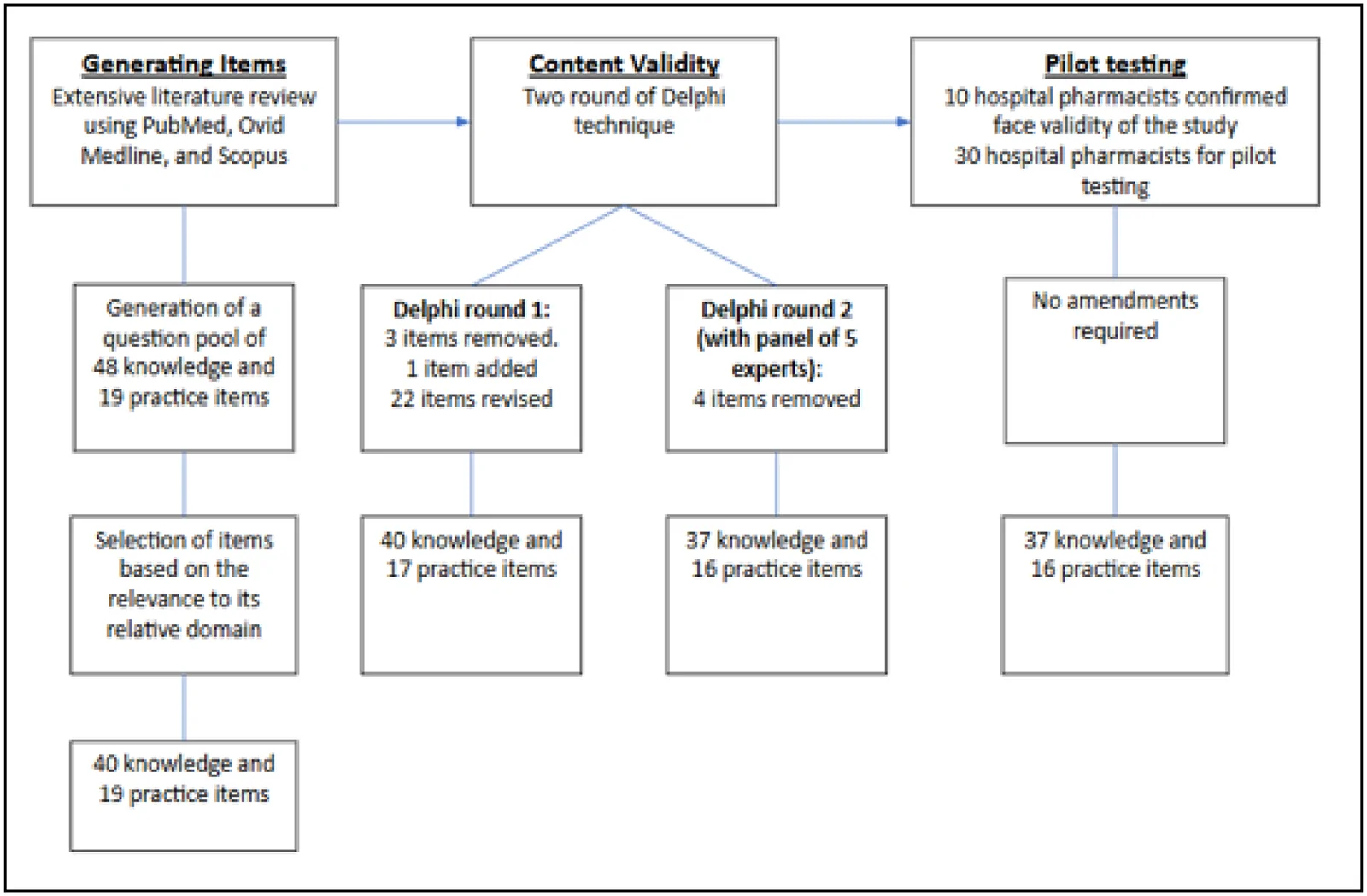
Summary of the stages in Phase I: Questionnaire Development.

Presented in Fig 1, an initial total number of 67 items was generated, then filtered after content validity and pilot testing to a final list of 53 items.

#### 2.5.2. Data collection.

Participants were recruited using non-probability sampling, including snowball and convenience sampling techniques, which ensured the inclusion of individuals from different demographics. A token of appreciation given to the first 50 participants in the form of an RM20 Touch’ N Go e-Wallet voucher. Survey Monkey is utilized because it provides faster distribution of surveys, accurate data input, and allows exporting the collected data in various formats [18].

Emails, LinkedIn, and social media, including Instagram, were used to post the survey poster and to contact the pharmacists’ representative in hospitals across Malaysia, identified through the hospital websites, to aid the dissemination of questionnaires. Participants who met the criteria were invited to take part in the study via a survey link. The purpose of the questionnaire and explanatory statement was incorporated into the survey to ensure that informed consent was provided. Participants agreed by accepting the consent statement and selecting “Yes” before completing the data collection form. Information regarding the confidentiality of personal data and the data collection process was clearly explained.

### 2.6. Sample size

A sample size of 50–100 respondents was targeted to be recruited. According to several studies regarding sample size estimation for questionnaires, to ensure validity and reliability of data analysis, at least 30 participants are required for reliability, and at least 50 for validity. Given this, recruiting at least 50 respondents can help to achieve statistical accuracy and for the detection of significant relationships [19–21]. A total of 105 participants were invited to take part in the study, and responses were received from 68 individuals (Response rate:64.8%).

### 2.7. Phase II-Data Analysis: Construct Validity & Reliability testing

Collected responses were analysed using the Statistical Package for the Social Sciences (SPSS) and R software, statistical programs used for data analysis. Descriptive statistics were used to summarise participants’ demographic information, such as age, gender, and education level.

**Fig 2:**
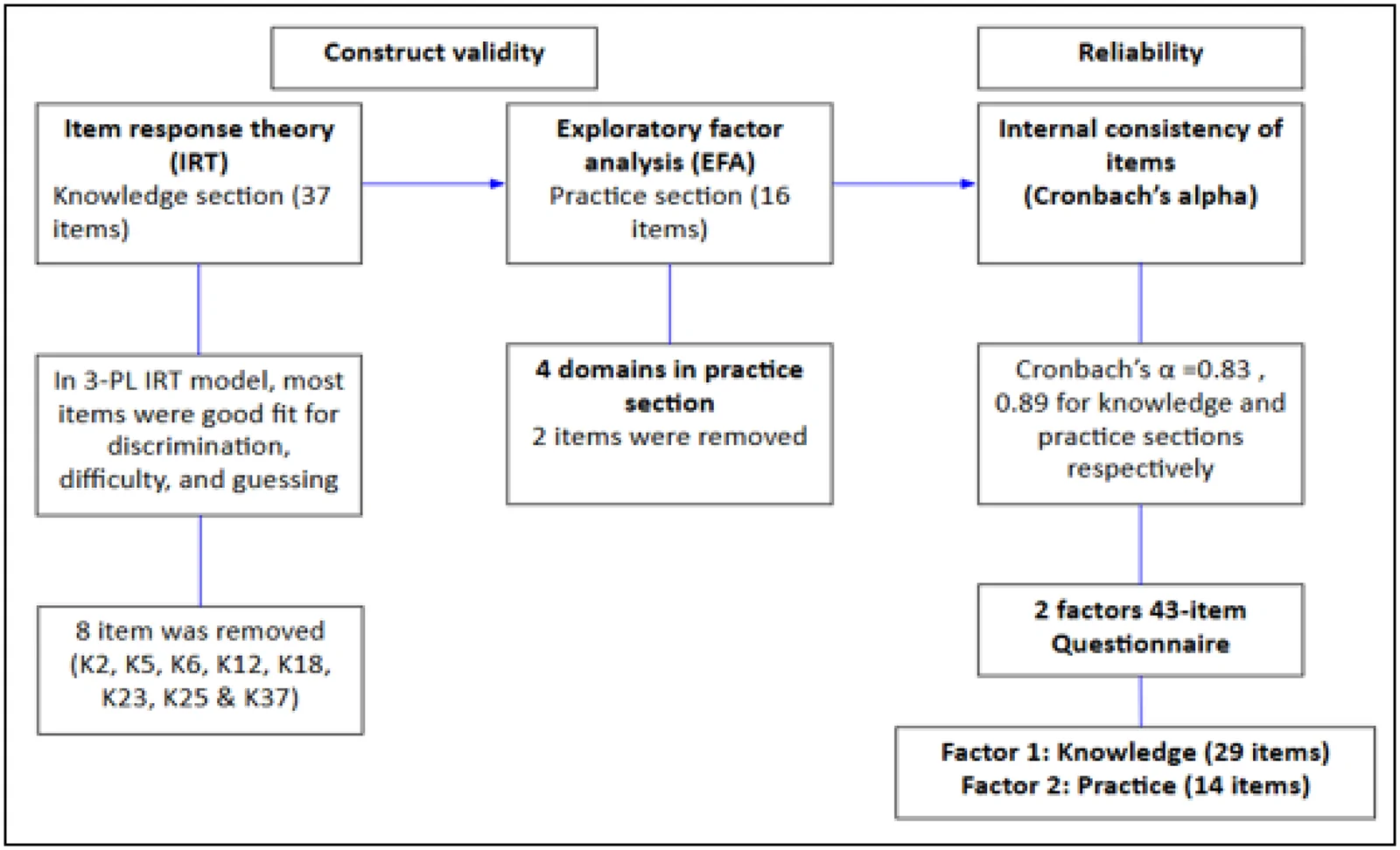
Construct validity and reliability of the questionnaire.

To assess construct validity, the 3PL-item response theory (IRT) model and exploratory factor analysis (EFA) were applied for the knowledge and practice domain, respectively Fig 2. IRT evaluates how responses are influenced by both the traits of the individual respondents and the questionnaire items’ characteristics [22]. The quality of items is measured by 3 parameters: item discrimination (a), difficulty (b), and guessing (c). Item difficulty determines how the item behaves along different ability scales, item discrimination determines how well items differentiate individuals with different traits, whilst guessing item determines the probability of a low-ability responder to give a correct answer due to random guessing [23]. Acceptable cut-off values for the parameters were above 0.25 for item discrimination, between −3–3 for item difficulty, and below 0.4 for item guessing [24,25]. EFA was used to identify the underlying factor structure, examining the relationships between items about how many factors exist or which items correspond to which factors [26]. Factor loading value greater than 0.4 will be accepted [27].

To assess reliability, Cronbach’s Alpha was used to measure internal consistency of the items [28] Values range from 0 to 1, and a high Cronbach’s alpha could indicate consistent responses across related items, suggesting they measure the same concept. The value of 0.60 and above is considered acceptable for the items to be reliable [25].

### 2.8. Ethics approval

Monash University Human Research Ethics Committee approved this study (MUHREC-46112). The research was conducted in accordance with the ethical principles outlined in the Declaration of Helsinki. The purpose of the study, prospective risks and benefits, and participants’ anonymity were explained by the researcher. Participants were given an explanatory statement, and informed consent was obtained. The confidentiality was maintained by assuring that no identifying demographic information existed. Participants were informed that the data would be anonymised and no identities would be collected. Informed consent was obtained from all participants who were involved in the study. Participants agreed by accepting the consent statement and selecting “Yes” before completing the data collection form.

## 3. Results

### 3.1. Participants’ sociodemographic characteristics

68 participants were recruited with a mean age of 31.25 years. The results revealed that the majority of them were female (77.9%), held a Bachelor’s degree (80.9%), and graduated from a private university (67.6%). A mean of 7.13 years of working experience among the pharmacists, and mostly are working in the ward (33.8%). The majority have received training on AMS (64.7%); however, a substantial proportion have not received training on antimicrobial prophylaxis among cancer patients (83.8%). In Table 1, the study participants were described in terms of their education, experience, and job scope in the hospital, ensuring that the findings remain relevant and applicable to healthcare professionals in this field.

**Table 1:** Socio-demographic characteristics of participants (n = 68).

Variables	Mean (SD)	Frequency and Percentage (n; %)
**Age**	31.25 (5.13)	
**Gender**		
Male		15 (22.1)
Female		53 (77.9)
**My qualification in pharmacy is _____.**		
Bachelor’s degree		55 (80.9)
Master degree		13 (19.1)
**I graduated from a _____ university.**		
Government		22 (32.4)
Private		46 (67.6)
**I have _____ years of working experience as a pharmacist**	7.13 (5.103)	
**I’m currently working as a_____ pharmacist**		
Inpatient		15 (22.1)
Outpatient		11 (16.2)
Ward		23 (33.8)
Other		19 (27.9)
**Do you have experience working as a pharmacist in the oncology ward/unit?**		
Yes		23 (33.8)
No		45 (66.2)
**Have you ever received training about antimicrobial stewardship (AMS)?**		
Yes		44 (64.7)
No		24 (35.3)
**Have you ever received training about antimicrobial prophylaxis among cancer patients?**		
Yes		11 (16.2)
No		57 (83.8)

### 3.2. Item response theory (IRT)

The analysis of the knowledge section of the questionnaire using the 3-PL IRT model is summarized in Table 2. The sub-domains of the knowledge section are infections in cancer patients (K1-K14), AMS and AMR (K15-K20), pharmacists’ role (K21-K24), prevention of infection in cancer patients (K25-K36), and patient safety and error prevention in antimicrobial prophylaxis use (K37). Item retention followed a predefined hierarchy prioritizing discrimination (a ≥ 0.25) and difficulty (−3 to +3) as mandatory exclusion criteria. In contrast, pseudo-guessing (c ≤ 0.4) and goodness-of-fit (p > 0.05) were treated as secondary diagnostics. Items may be retained to preserve the tool’s content validity as they represent critical clinical sub-domains. This prioritization of clinical relevance over strict statistical fit ensures the questionnaire maintains comprehensive construct coverage [29].

**Table 2:** Results of the IRT analysis in the knowledge section (n = 37), by using the 3-Parameter Logistic (3-PL) model.

Items	Item discrimination (a)	Item difficulty (b)	Guessing (c)	*x*^2^(df = 7)	P values
K1: Cancer type, history of infections, myelosuppressive treatments are not the factors that affect infection risk among cancer patients.	14.35	1.22	0.00	0.74	1.00
K2: Infection is a major cause of morbidity and mortality of cancer patients.	−22.32	0.70	0.83	0.21	1.00
K3: There is no association between tumor stage and the risk of infection.	10.55	1.53	0.06	6.53	0.48
K4: Gram-positive bacteria is the most common pathogen found in bacterial bloodstream infections in patients with malignancy.	3.95	0.13	0.27	13.26	0.07
K5: E.coli is the most common gram-negative bacteria found in bacterial bloodstream infections in cancer patients.	0.66	−3.13	0.00	13.59	0.06
K6: Patients with cancer have a higher risk of getting C.difficile infection (CDI) compared to non-cancer patients.	−28.35	1.32	0.52	0.12	1.00
K7: Cancer patients with previous latent infection of Hepatitis B will not have risk of neutropenia following chemotherapy treatment.	3.86	1.57	0.00	0.48	1.00
K8: HIV-negative cancer patients have a poorer prognosis of Pneumocystis jirovecii pneumonia than HIV-positive patients.	1.43	0.92	0.00	5.60	0.59
K9: Solid tumour patients are more susceptible to the reactivation of Hepatitis B virus and Hepatitis C virus than hematological malignancies patients.	2.02	0.49	0.00	3.14	0.87
K10: There is no risk of viral infection among cancer patients undergoing chemotherapy.	15.12	1.82	0.00	1045.86	0.00
K11: Reactivation of latent infection like herpes simplex virus (HSV) is uncommon in cancer patients undergoing chemotherapy.	15.82	1.88	0.07	6.44	0.49
K12: Antifungal prophylaxis can directly affect the morbidity and mortality of cancer patients related to invasive fungal infections (IFIs).	0.32	−7.64	0.07	7.58	0.37
K13: There are no known risk factors for multidrug-resistant infections in cancer patients.	31.48	1.33	0.00	0.02	1.0
K14: Antifungals should never be given to palliative care patients due to the adverse effects, even if it is needed for symptom relief.	1.66	1.69	0.00	3.06	0.88
K15: Antimicrobial prophylaxis is required for all cancer patients regardless of the risk of infection.	3.16	0.75	0.00	1.57	0.98
K16: Antimicrobial stewardship can increase the risk of insufficient antimicrobial coverage and cause detrimental effects to the cancer patients.	2.04	1.26	0.00	3.34	0.85
K17: Cancer patients who require multiple courses of antibiotics for prevention of infection should take it for a longer duration as this would not cause resistance.	3.08	1.36	0.00	3.44	0.84
K18: There is an increasing trend of resistance of candida species towards fluconazole and itraconazole.	−0.28	8.72	0.09	5.96	0.54
K19: Antimicrobial stewardship can improve patient outcomes and reduce risk of antimicrobial resistance. It is unnecessary to consider this aspect when initiating antimicrobial prophylaxis for cancer patients.	1.60	0.89	0.00	7.33	0.40
K20: Cancer patients with febrile neutropenia are recommended to receive empirical vancomycin for 48–72 hours to ensure efficacy while reducing the risk of antimicrobial resistance.	25.15	0.80	0.35	5337.38	0.00
K21: Risk assessment of chemotherapy-induced neutropenia is not the role of pharmacy.	18.74	1.19	0.05	11.29	0.13
K22: Oncology pharmacists are solely involved in the medication management of antimicrobial prophylaxis and are not involved in medication adherence assessment and side effect counselling.	31.56	1.33	0.00	12.75	0.08
K23: Pharmacists must always refer to established guidelines such as International Society of Oncology Pharmacy Practitioners (ISOPP), British Columbia Cancer Agency (BCCA), and evIQ.	−5.36	0.15	0.96	3.80	0.80
K24: Nurses do not suggest adjustments to oncology treatment plans, which is a responsibility handled by clinical pharmacists.	9.34	1.18	0.47	10.00	0.19
K25: Relevant serologic tests such as HIV, Hepatitis B virus (HBV), Hepatitis C virus (HCV), and Cytomegalovirus (CMV) are recommended to guide the prophylactic regimen.	−5.49	0.22	0.96	3.50	0.84
K26: Antiviral prophylaxis for Herpes Simplex Virus is not necessary if patients have been given Cytomegalovirus prophylaxis with Ganciclovir.	4.82	1.46	0.55	7.71	0.36
K27: Azoles as an antifungal prophylaxis in cancer patients, can be administered together with drugs used in cancer therapy	24.91	0.99	0.53	17.87	0.01
K28: Antifungal prophylaxis is recommended in every patient with neutropenia due to chemotherapy.	2.23	0.17	0.00	7.95	0.34
K29: Quinolone antibiotic is rarely used in cancer patients with chemotherapy-induced neutropenia.	0.33	−1.05	0.02	11.34	0.12
K30: Itraconazole, as a prophylaxis to prevent bacteremia, is used in acute lymphoblastic leukemia (ALL) pediatrics during induction therapy.	16.90	0.41	0.26	6.33	0.50
K31: Trimethoprim/sulfamethoxazole should not used as an antibiotic prophylaxis for PJP infection as it is reserved for PJP treatment	16.70	1.25	0.07	11.50	0.12
K32: Vancomycin metabolism and excretion will not be altered in cancer patients with worsening renal function from chemotherapy.	8.33	1.20	0.00	0.29	1.00
K33: Pharmacists can create a management plan to prevent neutropenic complications solely based on chemotherapy-induced neutropenia risk.	0.59	−1.75	0.01	11.20	0.13
K34: Cancer patients are recommended to take all vaccinations to prevent infection.	3.88	1.65	0.45	9.79	0.20
K35: Valacyclovir with the dose of 500 mg BD is given for all cancer patients with HSV.	4.76	0.81	0.24	7.28	0.40
K36: Antifungal prophylaxis should be continued until neutrophil count recovers.	1.07	−0.79	0.00	22.46	0.00
K37: Antimicrobial prophylaxis given through invasive routes such as the intravenous route of administration may pose risk of infection in cancer patients.	0.06	−12.67	0.17	9.04	0.25

As the acceptable range for the item discrimination (a) is 0.25 and above, most of the knowledge items were within the acceptable range; however, items K2, K6, K18, K23, K25, and K37 were below 0.25, and thus removed from the questionnaire.

The difficulty (b) for most items fell within the acceptable range of −3–3, except for items K5, K12, K18, and K37, which were removed from the questionnaire.

The acceptable range for the items’ guessing (c) is 0.4 or below. K2, K6, K23, and K25 items were out of range for the guessing, so removed from the finalised questionnaire. Items in the questionnaire will not be removed solely for exceeding the guessing threshold, provided they meet the criteria for item discrimination and difficulty.

Regarding item-goodness-of-fit consideration, only 4 items (K10, K20, K27, and K36) have a p-value of less than 0.05, while the rest exceed the significance level. However, the remaining items were retained in consideration of the items’ relevance and benefits for the assessment tool’s objective.

### 3.3. Exploratory factor analysis (EFA)

Through SPSS, principal analysis suggested a 4-factor model extraction based on the intercept point on the scree plot with eigenvalue criterion (≥1). This aligns with the number of domains in our practice section, shown in Table 3. Through the Varimax rotation method, all of the 16 items in the practice domain have an acceptable factor loading of >0.4, indicating their high correlation within their respective factor. Hence, all the practice items were retained. The KMO of 0.819 and Bartlett’s Test of Sphericity (p < 0.001) indicate patterns of correlations between items able yield stable factors in an Exploratory Factor Analysis (EFA).

**Table 3:** Results of the EFA of the practice domain.

Factors	Items	Factor loading	Cronbach’s alpha	KMO and Bartlett’s Test
Role of pharmacist	P1: I counsel patients on oral cancer drugs according to the internationally recognised guidelines like eVIQ and International Society of Oncology Pharmacy Practitioners (ISOPP).	0.664	−0.13	0.819
	P2: I seldom counsel the patient’s caregivers on prevention and management of infection due to privacy issues.	0.648		
Antimicrobial prophylaxis in cancer patients	P3: I encourage cancer patients with neutropenia to do regular outdoor activities to strengthen their bodies and boost up their immunity.	0.599	0.76	
	P4: I recommend yearly influenza vaccination with live vaccine for all patients receiving chemotherapy for malignancy and all household family members.	0.795		
	P5: I recommend to do a serological status test before initiation of chemotherapy but it should not delay the initiation of chemotherapy.	0.670		
	P6: I do not recommend Entecavir prophylaxis for cancer patients with serologic status of HbsAg-negative and anti-Hbc-positive.	0.527		
	P7: I recommend to rule out HIV infection before starting Entecavir or Tenofovir as a HBV reactivation prophylaxis.	0.776		
	P8: I do not use systemic antibacterial prophylaxis for children whose therapy is not expected to result in severe neutropenia (absolute neutrophil count <500/μL) for at least 7 days.	0.611		
Management of drug-drug interaction between chemotherapy and antimicrobial prophylaxis	P9: I check on patients’ medication history including OTC medications, supplements, and herbal remedies prior to recommending antimicrobial prophylaxis.	0.787	0.80	
	P10: I do not take into account the potential drug-drug interaction of Azoles and Tyrosine kinase inhibitors because the benefit outweighs the risk.	0.709		
	P11: I will ensure there is sufficient time for the potential interacting cancer drug to be eliminated before initiating antifungal prophylaxis (e.g.,: Azoles)	0.704		
	P12: I recommend the concurrent use of azole antifungals to prevent the risk of antifungal infections while being on chemotherapy agent vincristine.	0.509		
	P13: I recommend the alternative use of amphotericin B for antifungal prophylaxis when there are interactions between first line azoles and other chemotherapy agents.	0.582		
Monitoring and follow-up of the antimicrobial prophylaxis	P14: I recommend starting TDM for fluconazole as antifungal prophylaxis due to its linear pharmacokinetics properties.	0.814	0.63	
	P15: I recommend doing a serology status test following HBV vaccination.	0.584		
	P16: I rarely recommend monitoring HBV DNA levels and liver biochemistry every 3 months in cancer patients taking antiviral therapy to prevent reactivation of Hepatitis B reactivation.	0.639		

The reliability of items in the domain of the role of pharmacist was −0.13, which is below the cut-off value of 0.6 [25], signifying that there is low internal consistency between items in this domain, so P1 and P2 are removed. The other domains of the practice section demonstrated high internal consistency.

### 3.4. Cronbach’s alpha

Considering the results of IRT and EFA, 8 knowledge and 2 practice items were removed from the questionnaire. The 43-item questionnaire demonstrated an overall Cronbach’s alpha of 0.77, indicating strong internal consistency among the items. The reliability testing of the knowledge items recorded a value of 0.83, while the practice items showed a similarly high internal consistency with a value of 0.89, as presented in Table 4.

**Table 4:** Internal consistency reliability of the final 43-item questionnaire.

Items	Cronbach’s alpha
Knowledge domain (29 items)	0.83
Practice domain (14 items)	0.89
Overall	0.77

## 4. Discussion

Numerous studies have examined pharmacists’ roles in antimicrobial prescribing and their involvement in antimicrobial stewardship interventions across a variety of clinical settings [30] However, these investigations have largely targeted general antimicrobial use rather than focusing specifically on antimicrobial prophylaxis in oncology. Cancer patients, who are particularly vulnerable to opportunistic infections and complex drug–drug interactions due to intensive anticancer regimens, remain underrepresented in this body of research. To the best of our knowledge, the present study is the first to directly address this gap by developing and validating a dedicated assessment tool to evaluate pharmacists’ knowledge and practice of antimicrobial prophylaxis in cancer care. The study establishes the content validity, construct validity, and reliability of the instrument, providing a foundation for future application among a wider population of pharmacists.

The psychometric property of the knowledge section in the questionnaire was measured by the three-parameter logistic (3PL) model. It is suitable as our knowledge items are multiple-choice questions where guessing can influence the response. This finding is consistent with Hassan & Mohammed, who also reported that a 3-parameter logistic model provides a more comprehensive assessment of item performance [31]. The majority of the knowledge items fall within an acceptable range of discrimination (a), difficulty (b), and guessing (c) parameters. However, 8 items (K2, K5, K6, K12, K18, K23, K25, K37) were removed due to the deviations that fall out of the acceptable range of the parameters, highlighting the high validity of the questionnaire, as improper items were removed. Among the 8 items, K5, K12, and K37 showed a low b value, which indicates that the questions are relatively easy. Possible reasons for these items to fall beyond the acceptable range could be due to clinical practices and common medical knowledge of the pharmacists on bloodstream infections, antifungal prophylaxis, and IV routes, which makes it easier to answer [32,33]. Item K18 exhibited a low a value and a very high b value, indicating that the question is difficult and has poor discrimination. This may be explained by the fact that Candida species vary widely in their susceptibility patterns to azole antifungals such as fluconazole and itraconazole, making antifungal resistance patterns complex and difficult to predict in practice. Recent surveillance and systematic analyses have documented increasing trends of fluconazole resistance among multiple Candida species, particularly non‑albicans isolates, underscoring the growing challenge of azole‑resistant candidiasis in clinical settings [34].

Items K2, K6, K23, and K25 similarly showed a low a value but with a high c value, indicating a high possibility for the poorly performed candidates to guess the correct answer. It is likely due to the generality of the items K2 and K6 on healthcare-associated infection mortality rate and opportunistic infections, respectively, increasing the guessing tendency [35]. While for item K23, the analysis can be justified by Item-Writing Flaws, like logical cues that can lead to low discrimination [36]. Clues in wording, such as “established guidelines”, may have a leading question effect. Item K25, the familiarity of pharmacists with serologic tests such as HIV and Hepatitis B virus in guiding prophylaxis contributed to the low discrimination and high guessing, as the question is within their baseline knowledge [37]. Overall, Cronbach’s alpha for the knowledge section is 0.83, reflecting a high internal consistency of items.

Refining questionnaires through iterative IRT-based revisions is well recognized in psychometric validation. For example, a previous study applied IRT in the development of a knowledge and attitude questionnaire on antibiotic use and resistance in Swedish dairy farms, where items that failed to meet model assumptions were excluded to improve measurement validity [38]. Similarly, Edelen & Reeve demonstrated that IRT can be applied to shorten a depression questionnaire while maintaining validity and reliability, thereby reducing response burden [39]. Together, these studies emphasize the importance of IRT not only for improving psychometric properties but also for enhancing the efficiency of clinical measurement tools.

EFA was used to analyse the practice items, whereby principal analysis suggested a 4-factor model extraction aligning with the number of domains built. This highlights the complexity of antimicrobial prophylaxis among cancer patients that is influenced by multiple factors such as the choice of prophylactic agents, interaction between chemotherapy and antimicrobial agents as well as monitoring parameters. This is consistent with previous studies that highlighted the multifaceted factors in tailoring antimicrobial prophylaxis among cancer patients and the potential harm from drug-drug interactions [40,41]. All of the 16 items in the practice section have an acceptable factor loading of >0.4, reflecting the high correlation between the items within their respective domains. The negative Cronbach’s alpha value of −0.13 in the domain of the role of pharmacist reflects the conceptual heterogeneity [42]. Item P1 measures the institutional protocols and resources which vary across hospitals. This makes it a subjective measure rather than a universal measure of a pharmacist’s role. The low value is reasonably justified as there is a limited number of items in the domain, with only 2 items. In a study, Hayashi and Yuan quoted the Spearman-Brown formula to show that the higher the number of items, the closer Cronbach’s alpha approaches one [43]. This indicates that more items in this domain are required. Hence, the items in the domain of the role of pharmacist were removed due to their low Cronbach’s alpha. The final 43-item questionnaire is deemed to be a reliable assessment tool, with an overall Cronbach’s alpha of 0.77, indicating strong internal consistency.

### 4.1. Strengths of the study

The study is the first to develop such an instrument to evaluate pharmacists’ knowledge and practice regarding antimicrobial prophylaxis in cancer patients. It ensured questionnaire relevance through content validity, face validity, and pilot testing before finalizing items for data collection. This validation process improved clarity and appropriateness. Additionally, construct validity and reliability were tested using SPSS and R, providing strong evidence for the questionnaire’s construct, ensuring reliable and accurate findings.

### 4.2. Limitations of the study

This study has several limitations. The cross-sectional design restricts the ability to evaluate changes in pharmacists’ knowledge and practices regarding antimicrobial prophylaxis over time. The relatively modest sample size also limits the extent to which the findings can be generalized, and future studies with larger cohorts would strengthen the validation of the tool [44]. Besides, recruitment was concentrated within Malaysian hospitals, which may not fully capture the diversity of practice settings across different healthcare systems. Finally, only exploratory factor analysis (EFA) was performed to assess construct validity; future research should incorporate confirmatory factor analysis (CFA) to further validate the factor structure of the instrument [45]. CFA can confirm whether the data fits a hypothesized measurement model based on theory or prior research and involves steps like defining constructs, specifying factors, and testing model fit with item loadings and indices like RMSEA, CFI [46].

From these findings, it is evident that the gap in the lack of an assessment tool for pharmacists’ knowledge and practice of antimicrobial prophylaxis in oncology has been filled. It provides a feasible instrument for future studies aimed at strengthening pharmacists’ knowledge and practices in this field. With this in hand, targeted strategies and educational interventions can be recommended to address the identified issues, ensuring pharmacists are better equipped to manage infection risks in cancer patients. In the long term, these improvements have the potential to strengthen clinical decision-making, reduce the likelihood of medication errors, and enhance adherence to evidence-based guidelines. In the end, these efforts will contribute to safer and more effective oncology care, lowering infection-related morbidity and mortality while promoting better overall patient outcomes. This study successfully links back to its original objective by providing a validated tool to assess pharmacists’ knowledge and practices on antimicrobial prophylaxis among cancer patients.

## 5. Conclusion

The assessment tool on the knowledge and practice of antimicrobial prophylaxis in cancer patients has been developed and validated through data analysis, providing valuable insights from hospital pharmacists to enhance oncology care. The final questionnaire comprises 43 items, including 29 knowledge items and 14 practice items, organized into 5 knowledge domains and 3 practice domains. Given that cancer patients are more vulnerable to opportunistic infections, this tool emphasizes the need for diligent evaluation and implementation of appropriate antimicrobial prophylaxis strategies for this patient population.
