# Supplementary material for: Development and psychometric evaluation of an assessment tool assessing Pharmacists’ knowledge and practice on antimicrobial prophylaxis among cancer patients
Source: PLoS One. 2026 Aug 24;21(8):e0356508. doi: 10.1371/journal.pone.0356508 (PMC13502697; doi:10.1371/journal.pone.0356508)
Supplement: Supplementary file 1 [file pone.0356508.s002.docx]

| Comments by examiners | Amendment made |
| --- | --- |
| Reviewer 1:  Justification of Sample Size and Psychometric Methods: while the authors provide references supporting a minimum sample size of 50 participants for validity testing, the application of 3PL Item Response Theory (IRT) and Exploratory Factor Analysis (EFA) typically requires larger samples to ensure model stability and parameter precision. | We appreciate the reviewer’s rigorous attention to our psychometric approach. We agree that larger samples will be better for 3PL IRT and EFA. While N=50 is small, literature suggests that EFA performance depends more on factor loading strength than on sample size alone. According to de Winter et al. (2009), EFA can yield reliable results with N < 50 if the data exhibits high communalities (> 0.80) and a small number of factors. We have added our Kaiser-Meyer-Olkin (KMO) value [0.819] and Bartlett’s Test of Sphericity (p < 0.001) to our result section (line 289-291) to demonstrate that our data was statistically factorable. Furthermore, while 3PL IRT models benefit from larger samples to enhance parameter precision, smaller samples have been used in preliminary scale development studies, provided that parameter estimates are interpreted cautiously (Sahin, A., & Anıl, D. 2017).  Through SPSS, principal analysis suggested a 4-factor model extraction based on the intercept point on the scree plot with eigenvalue criterion (≥1). This aligns with the number of domains in our practice section, shown in Table 3. Through the Varimax rotation method, all of the 16 items in the practice domain have an acceptable factor loading of >0.4, indicating their high correlation within their respective factor. Hence, all the practice items were retained. The KMO of 0.819 and Bartlett’s Test of Sphericity (p < 0.001) indicate patterns of correlations between items able yield stable factors in an Exploratory Factor Analysis (EFA). |
| Reviewer 2:  Clarity and Consistency in Item Removal Criteria.  The criteria for item retention and removal in the IRT analysis are not always applied consistently:  Some items with poor goodness-of-fit (p < 0.05) were retained based on “relevance,” whereas others were removed strictly based on parameter thresholds.  The rationale for overriding statistical criteria in favor of content relevance should be more clearly justified and standardized.  The authors should clarify: A predefined decision hierarchy (e.g., statistical performance vs. content importance); how clinical relevance was operationalized when retaining poorly fitting items. | Thank you for this insightful comment. We have revised our result part to standardize the item retention process by implementing a predefined decision hierarchy (line 250-255). This hierarchy prioritizes clinical necessity in cases where statistical fit is marginal, which is a recognized approach in clinical instrument development where the goal is practical utility.  The analysis of the knowledge section of the questionnaire using the 3-PL IRT model is summarized in Table 2. The sub-domains of the knowledge section are infections in cancer patients (K1-K14), AMS and AMR (K15-K20), pharmacists’ role (K21-K24), prevention of infection in cancer patients (K25-K36), and patient safety and error prevention in antimicrobial prophylaxis use (K37). Item retention followed a predefined hierarchy prioritizing discrimination (a ≥ 0.25) and difficulty (-3 to +3) as mandatory exclusion criteria. In contrast, pseudo-guessing (c ≤ 0.4) and goodness-of-fit (p > 0.05) were treated as secondary diagnostics. Items may be retained to preserve the tool’s content validity as they represent critical clinical sub-domains. This prioritization of clinical relevance over strict statistical fit ensures the questionnaire maintains comprehensive construct coverage [9]. |
| Reviewer 3:  Negative Cronbach’s Alpha in Practice Subdomain  The reporting of a negative Cronbach’s alpha (-0.13) for the “role of pharmacist” domain requires deeper methodological discussion:  - Negative alpha values usually indicate problematic item coding, reversed items, or conceptual inconsistency.  - The explanation attributing this solely to a low number of items is insufficient.  The authors should:  Confirm whether items were reverse-coded correctly.  Discuss whether the items truly represent a coherent construct. | We appreciate the reviewer’s rigorous attention to the negative Cronbach’s alpha (alpha = -0.13) in the "Role of Pharmacist" domain. We revised the discussion section (line 387-397) to further justify the negative value of Cronbach's alpha. It can be due to conceptual inconsistency as item P1 was designed to measure the pharmacists’ practice in using relevant resources in the oncology unit which varies across hospitals. This makes it a subjective measure rather than a universal measure to define pharmacist practice. Hence, the “Role of Pharmacist” domain had been removed due to the poor internal consistency.  The negative Cronbach’s alpha value of -0.13 in the domain of the role of pharmacist reflects the conceptual heterogeneity [41]. Item P1 measures the institutional protocols and resources which vary across hospitals. This makes it a subjective measure rather than a universal measure of a pharmacist's role. The low value is reasonably justified as there is a limited number of items in the domain, with only 2 items. In a study, Hayashi and Yuan quoted the Spearman-Brown formula to show that the higher the number of items, the closer Cronbach's alpha approaches one [42]. This indicates that more items in this domain are required. Hence, the items in the domain of the role of pharmacist were removed due to their low Cronbach’s alpha. The final 43-item questionnaire is deemed to be a reliable assessment tool, with an overall Cronbach’s alpha of 0.77, indicating strong internal consistency. |
| Reviewer 4.  Lack of Updated and Comprehensive Contemporary Literature  The Introduction and Discussion sections do not sufficiently reflect the most recent evidence (2024–2025) on antimicrobial prophylaxis in oncology, particularly regarding the evolving balance between infection prevention and antimicrobial resistance.  While the manuscript cites established guidelines and foundational studies, it largely relies on older literature, with limited incorporation of recent:  Systematic reviews and meta-analyses evaluating the benefits and risks of antimicrobial prophylaxis in cancer patients;  Contemporary data on antimicrobial resistance trends in oncologic and hematologic populations;  Updated international guideline statements emphasizing selective prophylaxis and antimicrobial stewardship. | We thank the reviewer for this valuable comment. In response, we have updated the Introduction (line 64-71) (line 81-84) and Discussion (line 354-357) sections to incorporate more recent literature (2020–2025) related to antimicrobial prophylaxis in oncology. Specifically, we added recent reviews evaluating the benefits and risks of antimicrobial prophylaxis in cancer patients, as well as contemporary studies reporting antimicrobial resistance trends in oncologic populations. Additionally, we incorporated systemic reviews emphasizing selective prophylaxis and antimicrobial stewardship. These additions strengthen the timeliness and clinical relevance of the manuscript.  Aggressive cancer therapies compromise the immune system by suppressing white blood cell production. Recent evidence indicates that neutropenia is a significant adverse event associated with both conventional and novel anticancer agents [2], and is frequently observed following chemotherapy in real-world cohorts, where it is linked to serious infections and adverse clinical outcomes [3]. This highlights the importance of antimicrobial prophylaxis in preventing opportunistic infections. Recent systematic review shows antibiotic prophylaxis effectively reduces infection rates in oncology settings; However, the increased risk of antimicrobial resistance necessitates a careful balance between prevention benefits and the implementation of antimicrobial stewardship [4].  However, inadequate pharmacist knowledge can lead to inappropriate antibiotic selection, delays in administration, and prolonged antimicrobial therapy. Elshenawy et al. conducted a cross-sectional descriptive study among pharmacists to evaluate their knowledge, attitudes, and perceptions regarding antibiotic resistance and antimicrobial stewardship in a UK NHS Foundation Trust [7].  Item K18 exhibited a low a value and a very high b value, indicating that the question is difficult and has poor discrimination. This may be explained by the fact that Candida species vary widely in their susceptibility patterns to azole antifungals such as fluconazole and itraconazole, making antifungal resistance patterns complex and difficult to predict in practice. Recent surveillance and systematic analyses have documented increasing trends of fluconazole resistance among multiple Candida species, particularly non‑albicans isolates, underscoring the growing challenge of azole‑resistant candidiasis in clinical settings [34]. |
